# Transient Amplitude Modulation of Alpha-Band Oscillations by Short-Time Intermittent Closed-Loop tACS

**DOI:** 10.3389/fnhum.2020.00366

**Published:** 2020-09-04

**Authors:** Georgy Zarubin, Christopher Gundlach, Vadim Nikulin, Arno Villringer, Martin Bogdan

**Affiliations:** ^1^Technical Informatics Department, Leipzig University, Leipzig, Germany; ^2^Department of Neurology, Max Planck Institute for Human Cognitive and Brain Sciences, Leipzig, Germany; ^3^Institute of Psychology, University of Leipzig, Leipzig, Germany; ^4^Centre for Cognition and Decision Making, Institute for Cognitive Neuroscience, National Research University Higher School of Economics, Moscow, Russia; ^5^Neurophysics Group, Department of Neurology, Campus Benjamin Franklin, Charité Universitätsmedizin Berlin, Berlin, Germany; ^6^Department of Cognitive Neurology, University Hospital Leipzig, Leipzig, Germany; ^7^Mind Brain Body Institute at the Berlin School of Mind and Brain, Humboldt-Universität zu Berlin, Berlin, Germany

**Keywords:** tACS, closed-loop, alpha, EEG, stimulation, neural oscillations

## Abstract

Non-invasive brain stimulation (NIBS) techniques such as transcranial alternating current stimulation (tACS) have recently become extensively utilized due to their potential to modulate ongoing neuronal oscillatory activity and consequently to induce cortical plasticity relevant for various cognitive functions. However, the neurophysiological basis for stimulation effects as well as their inter-individual differences is not yet understood. In the present study, we used a closed-loop electroencephalography-tACS(EEG-tACS) protocol to examine the modulation of alpha oscillations generated in occipito-parietal areas. In particular, we investigated the effects of a repeated short-time intermittent stimulation protocol (1 s in every trial) applied over the visual cortex (Cz and Oz) and adjusted according to the phase and frequency of visual alpha oscillations on the amplitude of these oscillations. Based on previous findings, we expected higher increases in alpha amplitudes for tACS applied in-phase with ongoing oscillations as compared to an application in anti-phase and this modulation to be present in low-alpha amplitude states of the visual system (eyes opened, EO) but not high (eyes closed, EC). Contrary to our expectations, we found a transient suppression of alpha power in inter-individually derived spatially specific parieto-occipital components obtained *via* the estimation of spatial filters by using the common spatial patterns approach. The amplitude modulation was independent of the phase relationship between the tACS signal and alpha oscillations, and the state of the visual system manipulated *via* closed- and open-eye conditions. It was also absent in conventionally analyzed single-channel and multi-channel data from an average parieto-occipital region. The fact that the tACS modulation of oscillations was phase-independent suggests that mechanisms driving the effects of tACS may not be explained by entrainment alone, but rather require neuroplastic changes or transient disruption of neural oscillations. Our study also supports the notion that the response to tACS is subject-specific, where the modulatory effects are shaped by the interplay between the stimulation and different alpha generators. This favors stimulation protocols as well as analysis regimes exploiting inter-individual differences, such as spatial filters to reveal otherwise hidden stimulation effects and, thereby, comprehensively induce and study the effects and underlying mechanisms of tACS.

## Introduction

Non-invasive brain stimulation (NIBS) technology has gained increasing attention in the last few years from the scientific community (Bergmann et al., 2016; Antal et al., 2017; Thut et al., 2017; Vosskuhl et al., 2018), clinical (Palm et al., 2014; Yavari et al., 2017), sports (Edwards et al., 2017; Angius et al., 2018), military (Nelson et al., 2016), and other fields. One of the reasons for this growing interest is the successful modulation of cognitive, motor, and perceptual functions in numerous studies in different domains such as motor function (Feurra et al., 2011a; Brittain et al., 2013; Angius et al., 2018), visual (Zaehle et al., 2010; Helfrich et al., 2014), auditory (Riecke et al., 2015), somatosensory (Feurra et al., 2011b; Gundlach et al., 2016, 2017), or linguistic processing (Riecke et al., 2018; Wilsch et al., 2018) and for higher cognitive functions such as decision making, creativity, or self-aware dreaming (Sela et al., 2012; Voss et al., 2014; Lustenberger et al., 2015). Another reason is the widespread availability of various experimental, clinical protocols, and instructions (Bergmann et al., 2016; Antal et al., 2017; Tavakoli and Yun, 2017). Also, NIBS is generally a safe and well-tolerated form of brain stimulation with a comparatively simple set up. Transcranial alternating current stimulation (tACS) also has broad applications due to its ability to modulate ongoing neural oscillatory activity flexibly by precisely tuning stimulation parameters (such as frequency, phase, amplitude, or a combination of these) to each individual or each experimental session (Herrmann et al., 2013; Reato et al., 2013).

The advantages of tACS may be exploited even further in case of an adaptive or closed-loop approach, when stimulation parameters are tuned online during the experiment in a particular determined manner (Karabanov et al., 2016; Zrenner et al., 2016). In such a framework, brain responses to the stimulation, usually obtained from electroencephalography (EEG) or magnetoencephalography (MEG) data, serves as feedback and are used for the modification of control parameters. Significant efforts in recent research have been devoted to the establishment and application of closed-loop tACS-EEG/MEG models (Bergmann et al., 2016; Thut et al., 2017). However, despite an increasing number of proposed models, the field of adaptive tACS still lacks experimentally validated solutions. This can be explained by the complexity of the task: implementation and utilization of closed-loop tACS have various technical challenges and fundamental questions, which narrow and delay the development of this field.

First of all, despite numerous studies, the exact neural mechanisms of the effects of tACS are still not well understood. Animal, as well as human and computational modeling studies, suggest that weak alternating electric fields modulate spiking patterns of neurons utilizing neural entrainment (Deans et al., 2007; Fröhlich and McCormick, 2010; Ozen et al., 2010; Helfrich et al., 2014) or induction of spike-timing plasticity (Zaehle et al., 2010; Polanía et al., 2012; Vossen et al., 2015; Sliva et al., 2018). As suggested by resonance theory, these effects should be highly frequency-dependent and observable online during stimulation (Hutcheon and Yarom, 2000). In line with this, various studies have reported online effects of tACS on behavior (Kanai et al., 2010; Strüber et al., 2013; Vosskuhl et al., 2015; Gundlach et al., 2016) as well as markers of neural activity in the EEG, MEG or fMRI (Cabral-Calderin et al., 2015; Neuling et al., 2015; Witkowski et al., 2016). However, it has recently been shown (Asamoah et al., 2019) that effects of tACS on the motor system are, at least, partly related to transcutaneous stimulation of peripheral nerves in the skin beyond transcranial stimulation of cortical neurons (however, see Kasten et al., 2019; Krause et al., 2019; Vieira et al., 2019). Additionally, as suggested in some studies, processes leading to online effects of tACS and those leading to offline effects that sustain (or even manifest) after stimulation may rely on different neural mechanisms (Reato et al., 2010; Strüber et al., 2015). In a recent review article on the immediate effects of tACS (Liu et al., 2018), five possible neural mechanisms were suggested: “stochastic resonance, rhythm resonance, temporal biasing of neuronal spikes, entrainment of network patterns, and imposed patterns.” Importantly, how these mechanisms contribute to observed effects specifically and how they interact or compensate each other is largely unknown. Examining stimulation mechanisms, therefore, remains an open question.

Second, one of the biggest challenges with tACS and NIBS, in general, is high inter- and intra-individual variability of effects (Ziemann and Siebner, 2015). As mentioned by Guerra et al. (2017), this variability can be due to different factors, including physiological (variability of brain morphology, endogenous states, and different responses to stimulation), technical (particular setup and parameters of stimulation), and also statistical differences (numbers of participants and trials per groups and conditions). Tailoring stimulation protocols to individual factors may help to address (some) aspects of this variability.

Third, while behavioral effects for short-interval trial-by-trial alternated tACS points towards a fast onset of stimulation effects in the range of seconds (see Joundi et al., 2012), temporal dynamics of stimulation effects are largely unknown. The analysis of online stimulation effects is additionally impeded by tACS-induced artifacts in EEG data for instance that exceed the measured neural signals by several orders of magnitude. While some solutions to this challenge have been proposed (Witkowski et al., 2016; Noury and Siegel, 2017; Kasten et al., 2018; Kohli and Casson, 2019), the estimation of temporal dynamics of tACS-effects largely relies on probing lower temporal boundaries. Previous studies using conventional tACS (Vossen et al., 2015), found that intermittent tACS in the alpha range, applied over the visual cortex with trains of 8 s, but not 3 s, led to a modulation of visual alpha amplitude. Strüber et al. (2015) also applied tACS intermittently with 1 s intervals but found no significant after-effects. Given that a likely neural mechanism for tACS effects relies on the entrainment of ongoing neural oscillations by tACS, the relation between parameters such as phase, amplitude, and frequency of the targeted neural oscillations and applied tACS signal should modulate the effects of tACS, even more so for short stimulation intervals. By probing the coupling between tACS and alpha-band oscillations tACS effective may be rendered effective, even for low stimulation durations previously found ineffective. This would allow elucidating potential stimulation mechanisms as well as lower temporal boundaries of tACS effects.

In this work, we present the results of a study using a previously used closed-loop EEG-tACS protocol (Zarubin et al., 2018) by which the stimulation signal was phase coupled with ongoing alpha oscillations. In particular, we investigated the effects of repeated short durations of tACS (1 s) applied over the visual cortex on alpha amplitude when tACS signals and ongoing alpha visual alpha oscillations were phase-synchronized (in-phase) or in opposite phase (anti-phase) and in different states, when alpha levels were high in amplitude (eyes closed, EC) or low (eyes opened, EO). We specifically expect higher pre-to post-stimulation increases in alpha amplitudes for tACS applied in-phase as compared to an application in anti-phase and this modulation to be present in low-alpha amplitude states of the visual system (EO), but not high (EC). A dependency of stimulation effects on the phase of the tACS signal would also point towards online entrainment of visual alpha oscillations by tACS as a candidate mechanism for tACS effects. In addition to tailoring tACS to each subject’s alpha frequency and phase, we also adapted the analysis regime to acknowledge inter-individual differences in the spatial pattern of stimulation effects. Specifically, we studied potential modulations of neural oscillations conventionally, using single-channel and multi-channel data, from the parietal-occipital region and contrasted it with data individually derived *via* the application of spatial filters with the common spatial patterns approach (Blankertz et al., 2008). We expected pre- to post-stimulation changes in alpha-band activity to be more pronounced for individual spatial patterns.

## Materials and Methods

### Participants

Twenty healthy adults (nine females, mean age 28.4 ± 3.2 years) took part in this experiment and received monetary compensation for their participation. None of the participants had a history of psychiatric or neurological diseases and none were on any current medication affecting the central nervous system. Participants were informed about all aspects of the study and gave their written informed consent before the experiment. The study protocol was approved by the local ethics committee (“Modulation neuronaler Oszillationen mittels transkranieller Wechselstromstimulation und ihr Effekt auf die somatosensorische Wahrnehmung,” 12.08.2014, Reference number: 218-14-14072014). None of the participants have claimed that the feeling of stimulation was unpleasant, and none of them have experienced phosphenes.

### Experimental Procedure

**Figure 1:**
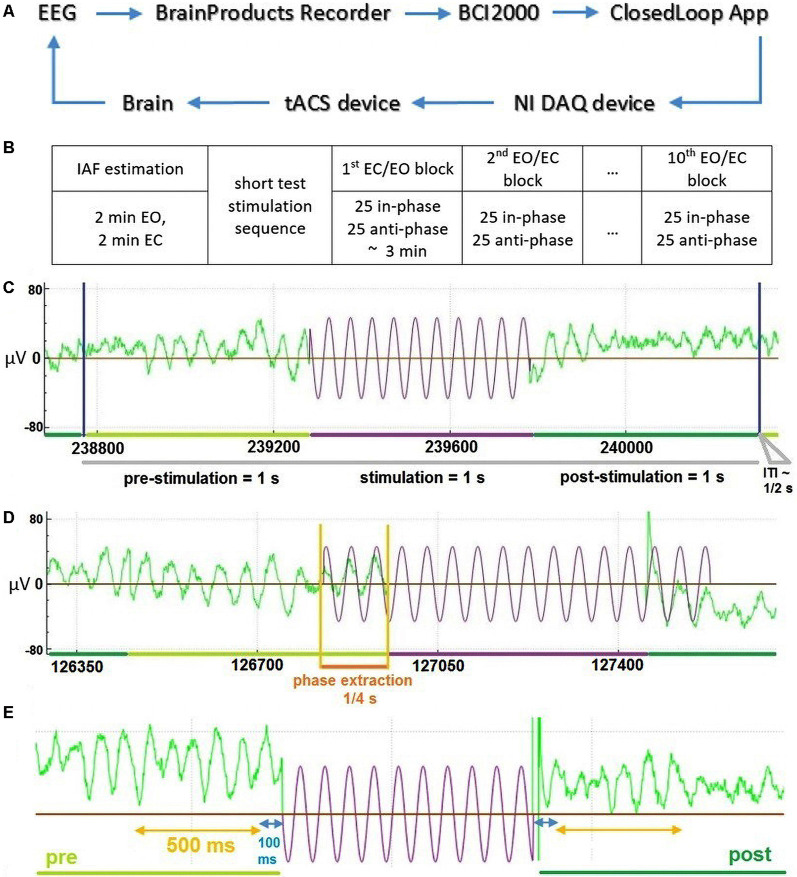
General description of the Closed-Loop model and the temporal structure of the data. **(A)** The general model scheme of the Closed-Loop setup. **(B)** Outline of experimental procedure. **(C)** An exemplary temporal trial structure with data in green representing recorded electroencephalography (EEG) data and data in violet depicting stimulation signal. **(D)** Illustration of the stimulation signal estimation based on an extraction interval of 0.25 s for an exemplary “in-phase” stimulation trial. **(E)** Temporal scheme of EEG data (green) used for the analysis of stimulation effects due to tACS (in violet—schematic representation of stimulation wave). Interval in blue represents 100 ms data interval excluded to reduced stimulation artifacts and potential edge filtering effect.

Each experimental session consisted of a preparation and information part, as well as the actual stimulation and EEG recording. During the preparation and information part, participants were introduced to the aim, set-up, and procedure of the study as well as the technical background of the stimulation. Individual contraindications for tACS were checked and the consent form was given, explained, and signed. tACS electrodes as well as EEG electrodes were then set up. Participants were subsequently instructed to sit relaxed, avoid movements, and later to keep their eyes opened or closed cued block-wise. The experiment included one session, which was completed by all participants. In the beginning, four 1-min resting-state EEG data (two EC, two EO, one after another) were collected to determine individual alpha frequencies (IAFs) by contrasting peaks in fast Fourier transform (FFT) spectra between EC and EO states at channel POz. Approximately five short stimulation sequences were then applied to test the correct functioning of closed-loop model units. Thereafter, 10 blocks (in an alternating sequence of EO and EC instruction) of 50 tACS-trials were executed consecutively with short breaks between each block. Each trial consisted of a 1 s pre-stimulation interval, 1 s of stimulation (with phase adjusted by prediction from pre-stimulation), a 1 s post-stimulation interval, and an inter-trial interval (ITI) (Figure 1C). The random ITI was in the range of 333–666 ms with a mean value of 500 ms. Every block consisted of 25 in-phase and 25 anti-phase stimulation trials in randomly shuffled order (Figure 1B). The total time for each block was 3 min, resulting in a total time of approximately 45 min for all 10 blocks with breaks between and a total stimulation time of 8 min, 20 s. This allowed us to examine changes in alpha-band activity based on the overall amplitude of alpha-band power/brain state (factor STATE: EO vs. EC) and the phase-relationship between ongoing alpha-band activity and the applied tACS signal (factor STIMULATION: in-phase vs. anti-phase).

### Electrical Stimulation

tACS electrodes (two conductive rubber 4 × 4 cm) were attached over standardized Cz and Oz channel locations (Jasper, 1958; Herwig et al., 2003) underneath the EEG recording cap and the sinusoidal alternating current was applied at IAF (calculated according to the procedure described above) using a battery-driven stimulator (DC-Stimulator Plus, NeuroConn, Ilmenau, Germany). Stimulation electrode positions were selected based on previous studies, in which modulations of visual alpha oscillations by tACS were reported (Neuling et al., 2013; Helfrich et al., 2014; Ruhnau et al., 2016). Impedances were kept below 10 kΩ with Ten20 conductive paste (Weaver and Company, Aurora, CO, USA). tACS at IAF was applied with an intensity of 1 mA (peak-to-peak) for all subjects. The stimulation signal for every trial was initially determined in a closed-loop application (custom made, C++), then generated through NI DAQ card (USB 6343, National Instruments, TX, USA) and transmitted to the DC-Stimulator Plus “remote input” port.

### EEG Recording

EEG data were recorded using Brain Products amplifier BrainAmp MRplus (Brain Products GmbH, Gilching, Germany) with 31 Ag-AgCl electrodes mounted in a passive EEG EasyCap using a standard 10-20 system layout without Oz and Cz electrodes, with reference and ground electrode positioned at FCz and AFz and applying a sampling rate of 500 Hz. The low sampling rate was used to reduce data transfer delays through components of the model, and during recording, the data was streamed from BrainVision Recorder through its RDA client to BCI2000 (open-source software, SchalkLab), which then transmitted it to the Closed-Loop application for analysis and optimal phase prediction.

### Closed-Loop Model

We used a closed-loop implementation based on a model described earlier (see Figure 1A and Zarubin et al., 2018) to apply tACS stimulation either in-phase or in anti-phase (phase-shifted by 180°) of ongoing visual alpha-band activity. In brief EEG signals were extracted online. For each stimulation interval, the phase of 250 ms of previous visual alpha-band activity recorded at electrode POz was estimated *via* its the Hilbert-transform. Depending on the experimental condition a stimulation signal of length 1 s was generated that was either in-phase with the previous 250 ms of data or in the opposite phase (see Figure 1D). This signal was then sent to the stimulator and applied as tACS either in or in the opposite phase. Any transduction delays were accounted for (please refer to Supplementary Materials for a detailed explanation).

### Data Analysis

#### Analysis of Alpha Power

The main focus of the analysis was to examine a potential modulation of alpha-band activity by tACS depending on: (1) the state (EO vs. EC); and on (2) the phase relationship of the applied tACS signal and ongoing visual alpha-band activity (in phase vs. opposite phase).

The 1 s long pre- and post-stimulation data intervals for each stimulation interval were extracted and bandpass-filtered with a 5–40 Hz 4th order Butterworth zero-phase filter, to reduce the impact of unspecific high-and low-frequency noise in our data. Pre-stimulation and post-stimulation alpha-band power were extracted from 500 ms before and 500 ms after each tACS interval (see Figure 1E; Note: we omitted the first 100 ms from the beginning of the post-stimulation interval, and the last 100 ms from the end of pre-stimulation to avoid any influence of the filtering edge effects and possible stimulation artifacts). For these windows, power values were then calculated *via* FFT of the detrended EEG data (zero-padding up to 512 samples). Afterward, individual alpha power values were calculated by averaging power values in the range of IAF − 1 Hz to IAF + 1 Hz (IAF was determined in the pre-experimental resting-state EEG measurement, as described above) for pre- and post-stimulation time windows and averaging across trials of each condition. Alpha-power values were extracted conventionally from a single parieto-occipital channel (POz; closest to stimulation electrode) and a parietal-occipital cluster (POC-region: P3, PO3, PO7, O1, POz, O2, PO8, PO4, P4). Also, we used a novel analysis approach tailored to account for inter-individual differences in signal representations by extracting alpha-signals, not from single channels but spatial filters weighing all electrodes to maximize signal representation (see Supplementary Materials for a detailed description). In brief, for each subject we calculated Common Spatial Patterns (first introduced by Blankertz et al., 2008), that either maximized pre- over post-stimulation alpha-power CSP(pre) or post-over pre-stimulation alpha-power CSP(post) for each trial with a cross-validation regime using all but the current trial for the calculation thereby avoiding overfitting of the data. Signals from these individual spatial filters were then used to calculate pre- and post-stimulation alpha power values for all experimental conditions as described above. Modulations of alpha power were tested with a repeated-measures ANOVA (ANOVA_RM_) comprising the factors TIME (pre- vs. post-stimulation), STATE (EO vs. EC) and STIMULATION (in-phase vs. anti-phase) separately for all signal sources: POz data, POC data, CSP(pre) data, and CSP(post) data.

#### Analysis of Average Power Spectra

To investigate the frequency specificity of alpha-power modulations, we computed and analyzed the FFT-derived power spectra separately for all experimental conditions, both time windows, and all signal sources. We additionally extracted the pre-experimental power spectra as the grand-mean FFT-spectra derived from 1 s long data segments for the EO and EC blocks of the resting-state measurement recorded from POz.

#### Analysis of Alpha Power Modulations Across the Time Course of the Experiment

In an exploratory *post hoc* analysis, we investigated whether the alpha power decreases found for the CSP(pre) data (see “Results” section) change across the time course of the experiment as responsiveness to the stimulation may change. For this purpose, pre-to post-stimulation power modulations in percent were calculated for each trial and averaged separately for each experimental block and condition. In an ANOVA_RM_ the factors BLOCK, STATE, and STIMULATION were then tested.

#### Analysis of Alpha Power Modulations Separately for Different Post-stimulation Time Windows

In a second *post hoc* analysis we wanted to investigate the time scale of post-stimulation alpha power decreases. For this purpose, we examined the temporal evolution of the decrease by comparing alpha power of a pre-stimulation time window [(−600 to −100) ms] to four different 500-ms long, overlapping, post-stimulation time windows [(100–600) ms, (200–700) ms, (300–800) ms, and (400–900) ms in relation to the onset of stimulation]. Pre-to-post modulations of alpha power values for the CSP(pre) data were modeled with an ANOVA_RM_ comprising the factors TIMEBIN, STATE, and STIMULATION.

Results for ANOVA_RM_ models were corrected for multiple comparisons using Bonferroni correction, when necessary. In the case of a violation of the homoscedasticity, degrees of freedom were corrected based on the Greenhouse–Geisser correction. Statistical analysis was performed in R (R Core Team, 2016), using the package afex (Singmann et al., 2018) running in R Studio (RStudio Team, 2015). Generalized Eta Squared (Bakeman, 2005) and Cohen’s d (Lakens, 2013) served as estimates of effect sizes. *Post hoc* contrasts and marginal means (Searle et al., 1980) were calculated *via* the emmeans package.

## Results

### Alpha Power Is Modulated by tACS in Individual Spatial Components

**Figure 2:**
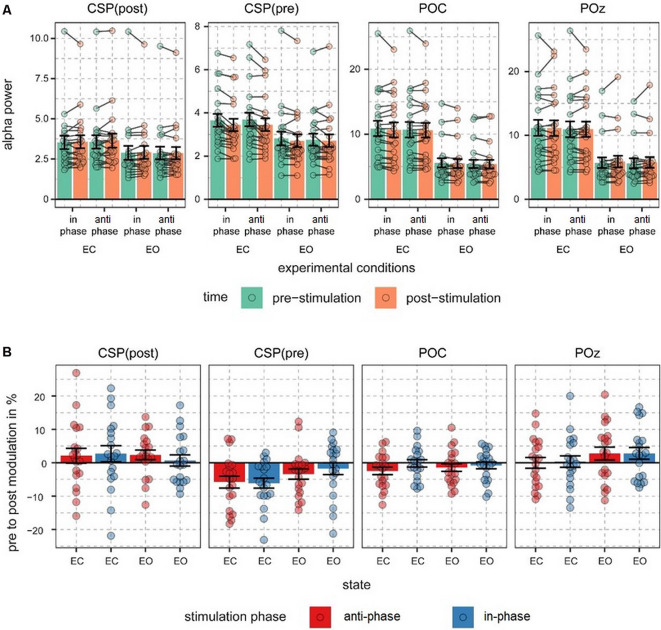
Alpha power values and pre- to post-stimulation modulations of alpha power. **(A)** Fast Fourier transform (FFT)-derived power values were calculated for a 500-ms long pre- and post-stimulation time window separately for all experimental conditions and signal sources. Note different scales. Connected dots represent single subjects’ pre- to post- alpha power changes. **(B)** Pre- to post-stimulation modulations in % are shown for all signals and conditions. Dots represent single-subject data and error bars represent standard error of the mean.

The main focus of the experiment was to analyze a potential modulation of visual alpha-band activity by the application of tACS either applied in- or anti-phase with ongoing visual alpha-band activity. As visible in Figures 2A,B, pre- and post-stimulation values only differ for power values derived from the CSP(pre) data. This difference is substantiated by the main effect for the factor time (*p* = 0.002; see Table 1): across experimental conditions pre-stimulation power values are larger [*M* = 3.23; CI = (2.48, 3.67)] than post-stimulation power values [*M* = 3.08; CI = (2.64, 3.82)]. Additionally, there is a trend for an interaction of factors TIME × STATE (*p* = 0.058), with larger pre- to post-stimulation decreases when eyes are closed (*M* = −0.23; SE = 0.047) as compared to EO (*M* = −0.076; SE = 0.047). As revealed by the main effects STATE, for all signals (*p*s < 0.001; see Table 1) alpha power values are always larger when eyes are closed as compared to when they are open.

**Table 1:** ANOVA_RM_ table representing the results of the analysis of FFT-derived power values separately for all signals.

signal	Factor	*df*	*F*	*p*	$\eta_{\text{G}}^{2}$
CSP(post)	TIME	(1,19)	0.524	>0.999	<0.001
	STATE***	**(1,19)**	**39.322**	**<0.001**	**0.037**
	STIMULATION	(1,19)	0.111	>0.999	<0.001
	TIME × STATE	(1,19)	0.647	>0.999	<0.001
	TIME × STIMULATION	(1,19)	0.025	>0.999	<0.001
	STATE × STIMULATION	(1,19)	1.636	0.8651	<0.001
	TIME × STATE × STIMULATION	(1,19)	0.709	>0.999	<0.001
CSP(pre)	TIME**	**(1,19)**	**17.14**	**0.0022**	**0.004**
	STATE***	**(1,19)**	**26.6**	**<0.001**	**0.09**
	STIMULATION	(1,19)	0	>0.999	<0.001
	TIME × STATE	(1,19)	7.246	0.0578	0.001
	TIME × STIMULATION	(1,19)	0.224	>0.999	<0.001
	STATE × STIMULATION	(1,19)	1.114	>0.999	<0.001
	TIME × STATE × STIMULATION	(1,19)	0.712	>0.999	<0.001
POC	TIME	(1,19)	3.684	0.797	0.002
	STATE***	**(1,19)**	**20.38**	**<0.001**	**0.12**
	STIMULATION	(1,19)	1.53	0.4588	0.001
	TIME × STATE	(1,19)	1.725	>0.999	<0.001
	TIME × STIMULATION	(1,19)	1.544	0.876	0.001
	STATE × STIMULATION	(1,19)	0.432	>0.999	<0.001
	TIME × STATE × STIMULATION	(1,19)	0.36	>0.999	<0.001
POz	TIME	(1,19)	1.3	>0.999	<0.001
	STATE***	**(1,19)**	**43.192**	**<0.001**	**0.253**
	STIMULATION	(1,19)	3.923	0.2492	<0.001
	TIME × STATE	(1,19)	0.347	>0.999	<0.001
	TIME × STIMULATION	(1,19)	0.015	>0.999	<0.001
	STATE × STIMULATION	(1,19)	0.226	>0.999	<0.001
	TIME × STATE × STIMULATION	(1,19)	0.166	>0.999	<0.001

Significant effects are marked by asterisks and bold text. ***p <0.001, **p < 0.01. *p*-Values are Bonferroni corrected for the four ANOVA_RM_ models.

**Figure 3:**
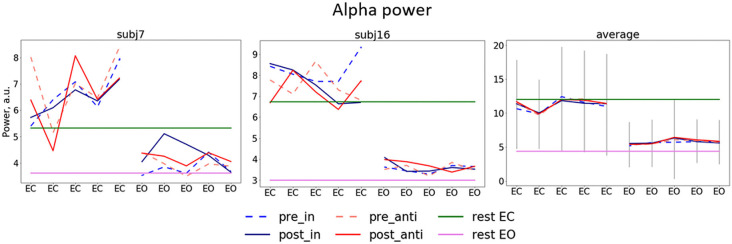
Dynamics of alpha power separate for stimulation blocks. Mean pre/post-stimulation values for in-phase and anti-phase relation (pre_in, post_in, pre_anti, post_anti) for exemplary subjects (the left and the center plots) and the group average (the right plot) first five blocks (eyes closed, EC) correspond to blocks in which participants had their EC and second five blocks eyes open (EO), green and pink horizontal lines represent alpha power for EC and EO in resting state, gray lines represent average standard deviation.

Alpha power values (conventionally) derived from the single electrode POz as well as the parieto-occipital cluster (POC), in contrast, are also modulated by the factor STATE (i.e., higher when eyes are closed as compared to EO; *p* <0.001), but are not significantly modulated by tACS or any interaction between the experimental factors (all *p*s > 0.292; see Table 1). For these channels single-subject signal dynamics vary substantially between subjects (largest between-subject variation when eyes are closed: average std = 6.806, mean = 11.25; for EO: average std = 3.817, mean = 5.742) with no clear changes between pre- and post-stimulation on the group level (see Figure 3).

Overall only for individual spatial components (see Supplementary Materials for individual and average topographical distributions of the weights), accounting for differences in the topographical distribution of modulated visual alpha-band activity, a decrease of visual alpha power was measurable, independent of the phase relationship between tACS and ongoing alpha-band activity.

### Modulation of Power of Neural Oscillations Is Specific to the Alpha Range

**Figure 4:**
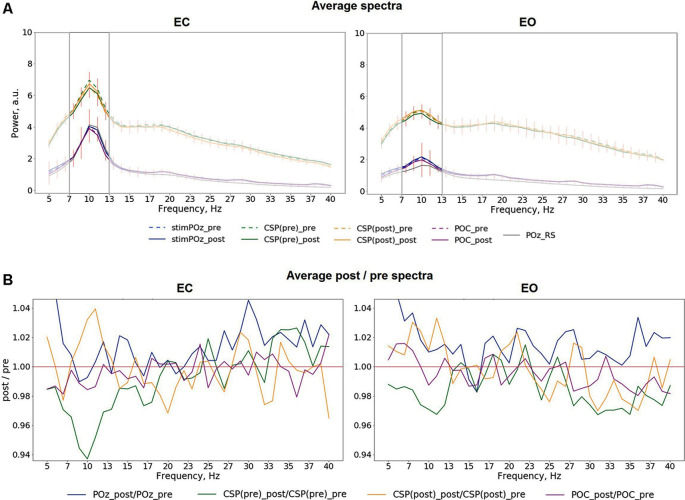
**(A)** Average pre- and post-stimulation spectra for different eye conditions, including both in- and anti-phase trials: EC (left), EO (right), red vertical lines represent the standard deviation of the average difference between pre- and post-stimulation. **(B)** Average relation of post-/pre-stimulation spectra.

Mean spectra averaged across all trials for different conditions, stimulation relations, with data from POz, both CSP components, POC, as well as relations of post- to pre-stimulation are shown in Figure 4. The analysis of the spectra revealed visible differences between pre- and post-stimulation time for signals extracted from both CSP components (Figure 4A) for when participants had their eyes closed as well as open. As visible in Figure 4B, these differences have their maxima in the alpha range without prominent changes to frequencies other than in a range near individual alpha bands.

### Modulations of Alpha Power Do Not Change Across the Time Course of the Experiment

**Figure 5:**
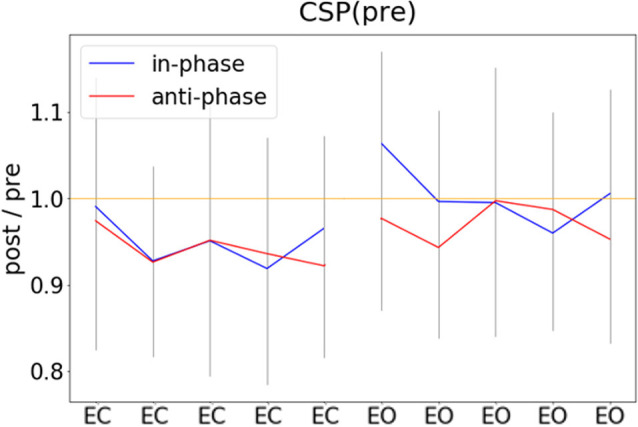
Pre- to post-stimulation power modulations computed separately for experimental blocks. Pre- and post-stimulation ratios of FFT-derived power values were calculated for a 500-ms long pre- and post-stimulation time window separately for all experimental conditions and experimental blocks, gray lines represent standard deviation.

In an exploratory *post hoc* analysis, we investigated whether the modulation of CSP(pre)-derived alpha power values by tACS changed across the experimental blocks. As visible in Figure 5, there was no systematic change of pre- to post alpha-power modulations across the time course of the experiment. Consequently, modeling CSP(pre)-derived pre- to post power modulations with an ANOVA_RM_ model revealed the factor BLOCK to be insignificant (see Table 2). Therefore, overall pre-to post-stimulation modulations of alpha power seemed to be stable across the experiment. Pre- to post-stimulation decreases were, however, dependent on the state, as revealed by the main effect for the factor STATE (*p* = 0.031) and was larger when eyes were closed [*M* = −5.786; CI = (−8.756, −2.816)] as compared to eyes open [*M* = −1.829; CI = (−4.8, 1.141)].

**Table 2:** ANOVA_RM_ table representing results of the analysis of FFT-derived pre-to post-stimulation power modulation for the CSP(pre) data for time-course analysis of the stimulation effects across the experiment.

Factor	*df*	*F*	*p*	$\eta_{\text{G}}^{2}$
STATE*	**(1,17)**	**5.563**	**0.0306**	**0.022**
STIMULATION	(1,17)	0.684	0.4196	0.002
BLOCK	(3.13, 53.2)	2.009	0.1213	0.021
STATE × STIMULATION	(1,17)	0.279	0.604	0.001
STATE × BLOCK	(3.33, 56.6)	0.289	0.8522	0.003
STIMULATION × BLOCK	(3.58, 60.9)	0.43	0.766	0.004
STATE × STIMULATION × BLOCK	(3.22, 54.7)	1.571	0.2043	0.017

Significant effects are marked by asterisks and bold text. *p < 0.05.

### Modulation of Alpha Power Is Transient

**Figure 6:**
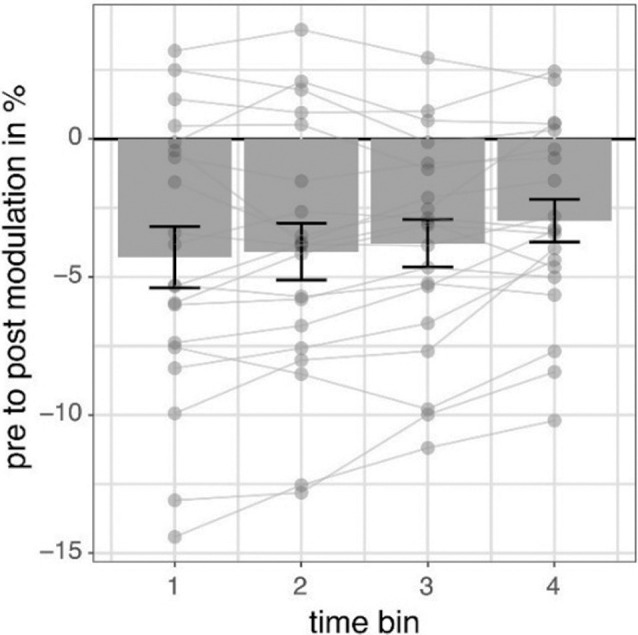
Pre- to post-stimulation power modulations separately for different time windows. The data represents pre- to post-stimulation modulations of alpha power from a 500-ms long pre-stimulation time window to four different 500-ms long overlapping post-stimulation time windows averaged for all experimental conditions in percent. Post-stimulation time bins are made up by data from the following time windows: (100; 600) ms, (200; 700) ms, (300; 800) ms, (400; 900) ms concerning the onset of the stimulation and always compared to the same pre-stimulation time window (−600; −100) ms. Error bars represent standard error of the mean and dots represent single subjects.

We investigated the time scale of post-stimulation alpha power decreases by additionally modeling the effects of pre- to post-stimulation power modulations separately for different overlapping post-cue time windows. The analysis revealed the factor TIMEBIN to be significant (*p* = 0.018; see Table 3). *Post hoc* linear contrasts revealed pre- to post-stimulation decreases to be modeled best by a linear decrease (*t*_(57)_ = 3.497; *p* = 0.001). Overall, independent of the experimental condition (i.e., EO vs. EC; stimulation in- vs. anti-phase) the pre-to post-stimulation decreases are largest right after the stimulation and then decay across subsequent time windows (see Figure 6).

**Table 3:** ANOVA_RM_ table representing results of the analysis of FFT-derived pre-to post-stimulation power modulation for the CSP(pre) data for analyzing the time scale of the stimulation effects.

Factor	*df*	*F*	*p*	$\eta_{\text{G}}^{2}$
STATE	(1,19)	3.201	0.0895	0.037
STIMULATION	(1,19)	0.58	0.4557	0.004
TIME BIN*	**(1.95, 37)**	**4.537**	**0.018**	**0.006**
STATE × STIMULATION	(1,19)	0.76	0.3941	0.007
STATE × TIME BIN	(1.34, 25.4)	1.303	0.2772	0.002
STIMULATION × BLOCK	(1.41, 26.9)	0.055	0.8926	<0.001
STATE × STIMULATION × BLOCK	(1.8, 34.2)	0.141	0.8487	<0.001

Significant effects are marked by asterisks and bold text. *p < 0.05.

## Discussion

Our study aimed to investigate the effects of tACS applied bilaterally over the visual cortex, tuned to neural alpha oscillations with a closed-loop EEG-tACS setup on visual alpha oscillations. Specifically, we have studied stimulation effects of tACS applied either in-phase or anti-phase with ongoing alpha oscillations during periods of a high-amplitude vs. low-amplitude alpha oscillations on the amplitude of alpha oscillations.

Overall, we found a decrease in alpha amplitude immediately after tACS when accounting for individual spatially specific alpha components with a cross-validation procedure. While these changes had an overall topographical center of gravity in occipital regions, they were individually specific and effects were not observable when data was extracted from a single occipital electrode or a general occipital electrode cluster as in a conventional analysis approach. Although the decreases in amplitude found for a 500-ms long time window seem to be only transient and attenuate across the range of 400 ms, they were constant across the time course of the experiment.

In contrast to previous studies, we found a decrease of alpha amplitude as a response to tACS. Zaehle et al. (2010) previously reported an increase of alpha amplitude after 10 min of tACS applied over occipital areas at the individual alpha frequency. Similarly, in various subsequently published studies the application of alpha tACS over visual areas in the range of minutes led to an increase in alpha amplitude (Neuling et al., 2013; Helfrich et al., 2014; Kasten et al., 2016). While these studies differ in their overall stimulation duration, some studies used intermittent short stimulation protocols closer to the design in our study. Strüber et al. (2015) used an experimental protocol similar to ours by intermittently applying 1-s long stimulation trials using conventional tACS. They found no evidence of a modulation of alpha power by tACS. However, they analyzed data only from a single channel (POz) and a longer time interval (1 s). Our data revealed significant stimulation effects to be present only in individual spatial components, but absent at POz and to be transient as they decreased within 400 ms after the end of stimulation. Vossen et al. (2015) applied longer stimulation durations with a different stimulation electrode montage (bilaterally over PO7/PO9 and PO8/PO10) and found that only 8-s intermittent stimulation, but not 3 s, led to pronounced alpha amplitude increases. In another study, Sliva et al. (2018) applied intermittent non-adaptive stimulation of a 6-s duration to investigate the influence of tACS on somatosensory perception and found that such stimulation was not sufficient to induce significant causal effects on EEG-measured alpha oscillations.

Because stimulation protocols with longer stimulation durations seem to lead to an increase in alpha amplitude and studies employing trains of stimulation with a duration of 3 s or less either found no evidence for a stimulation effect or a decrease in amplitude, as we did here, it is tempting to speculate about the parameters that shape the stimulation effect. If decreases and increases in amplitude represent two extreme cases, is there a stimulation duration that represents a transition from one to another? What additional factors may contribute to shaping the stimulation effects? Recent studies suggest that the brain state plays a crucial role: when eyes were closed or the room was not illuminated, alpha tACS did not lead to an increase in the amplitude, suggesting that tACS may not modulate the amplitude of oscillations that are already in a high amplitude state (Neuling et al., 2013; Ruhnau et al., 2016). An additional factor may be related to the electrode positioning. When in anti-phase and inter-hemispherically stimulating two coupled mu-alpha generators in the somatosensory system, we previously found a decrease of mu-alpha amplitude after a 5-min tACS application (Gundlach et al., 2017). This decrease was also found for inter-hemispheric tACS targeting theta oscillations (Garside et al., 2015). In a computational study, simulating stimulation after-effects in neural networks with nodes coupled with a time delay in-phasic stimulation led to amplitude increases, while anti-phasic stimulation led to no increases in oscillatory activity (Kutchko and Fröhlich, 2013). Therefore, further studies parametrically manipulating different factors such as duration and electrodeposition are required to map the effects of tACS more completely.

We found stimulation related decreases in the amplitude to be independent of the phase relationship between ongoing alpha oscillations and the tACS signal. Both in-phase, as well as anti-phase stimulation (i.e., stimulation phase being identical to the phase of ongoing alpha oscillation measured over POz vs. shifted by 180 degrees and thereby reversed in polarity), disrupted ongoing alpha oscillations. This finding is difficult to reconcile with a stimulation effect mediated by entrainment. While in animal studies online effects were found to be directly related to entrainment of ongoing neural activity by the applied electric oscillation (Fröhlich and McCormick, 2010; Ozen et al., 2010; Reato et al., 2010), in human studies investigating post-stimulation modulations of oscillations it was proposed that these offline effects may stem from LTP/LTD related effects (Zaehle et al., 2010; Vossen et al., 2015; Vosskuhl et al., 2018). Like others, we previously reported on tACS driven decreases in the amplitude of ongoing oscillations (Garside et al., 2015; Gundlach et al., 2017). While stimulation locations and protocols differed in these studies, the findings common to them and reported here showed, that amplitude decreases even beyond the stimulation period cannot be caused and explained by mere entrainment of ongoing oscillations by tACS on its own. Thus, it seems to be the case that offline effects are caused by neurophysiological mechanisms different from entrainment. Interestingly, similar to the effects found in the animal studies described above, the online effects of tACS measured in humans during the stimulation are consistent with the entrainment of neural activity by tACS. For instance, behavioral modulations depend on the stimulation frequency (Joundi et al., 2012; Santarnecchi et al., 2013) and phase of the tACS signal (Neuling et al., 2012; Gundlach et al., 2016). In the same vein in recent work by Fiene et al. (2020), it was shown that the interaction between ongoing stimulus processing and tACS was dependent on their phase relationship. The authors measured SSVEP amplitudes driven by a visual flicker after a period of tACS applied over visual areas with the same frequency as the flicker. Crucially they varied the phase relationship between tACS and flicker and found that the SSVEP amplitude varied accordingly. These findings show an interaction between stimulus processing and tACS modulated neural activity pointing towards a mechanism of entrainment of ongoing neural activity by tACS that affects stimulus processing even after the termination of the stimulation. It would be of great interest to see whether these effects would hold for other stimulation frequencies and could thus be related to the modulation of ongoing neural activity or whether these effects may arise from a mere phasic modulation of excitability. Overall, previous studies suggest that tACS effects may be caused by two different and potentially distinct mechanisms (see Heise et al., 2019): tACS may lead to online entrainment of ongoing oscillations and additional changes in neural plasticity responsible for stimulation outlasting offline effects. While Kar and Krekelberg (2014) found tACS-induced changes in neural adaptation to be potentially closely linked to changes in neural plasticity, the functional underpinnings of such effects as well as the relationship between online entrainment and offline neural plasticity remain unknown.

Interestingly, the stimulation effects in our study seem to be only transient, as they decreased after the end of the stimulation and were not different across the time course of the experiment (i.e., there was no evidence for an increased or decreased responsiveness to the stimulation). Effects related to neuroplastic changes seem to depend on the stimulation duration. For instance, neural excitability is longer modulated the longer tDCS was applied (Nitsche et al., 2003), and after-effects of tACS on behavior increased across the time course of the experiment (Heise et al., 2019). A tentative and an alternative interpretation of our findings showing the stimulation effect to be independent of the time in the experiment could be that the application of short stimulation periods in our experiment did not lead to plastic modulation of alpha generators, but instead briefly disturbed ongoing oscillations. Because alpha rhythm seems to fluctuate between different states of activity level (Freyer et al., 2009, 2011), the transient decrease in amplitude after the application of tACS may index a brief shift of alpha activity towards a lower activity level by tACS. However, when assuming online entrainment of alpha activity by tACS, it is puzzling that both in- and anti-phase stimulation lead to a similar effect of amplitude attenuation. Specifically, one would hypothesize a synchronous application (no phase difference and the same polarity) not to disrupt the specific oscillation, while an asynchronous application would be more likely to be disruptive. We extensively tested our setup and phase extraction as well as forecast algorithms to ensure that the relation of the stimulation phase and phase of ongoing alpha oscillations was captured correctly (see Zarubin et al., 2018). While small deviations in the phase relation may arise from the phase estimation, forecast process, and underlying assumptions of stationarity, the overall phase relation is thus estimated accurately. If in- and anti-phase stimulation are indeed different in their phase relationship, why would they lead to a similar effect, namely the decrease in alpha amplitude? Several recent studies have suggested that alpha oscillations measured on a macroscale-level are the product of different alpha generators with different spatial, laminar, or functional profiles rather than being produced by a single generator (Bollimunta et al., 2008; Haegens et al., 2015; Keitel and Gross, 2016; Scheeringa et al., 2016; Barzegaran et al., 2017; Benwell et al., 2019; Schaworonkow and Nikulin, 2019). The potential interaction, coupling, and interdependence of different alpha generators are, however, vastly unknown. There is some evidence that different alpha generators may be antagonistically coupled, for instance, seen in the relationship of visual alpha and sensorimotor mu-alpha (Gerloff et al., 1998; Neuper and Pfurtscheller, 2001) or the focal down- and surround up-regulation of alpha generators in the somatomotor system (Suffcynski et al., 1999) or the different alpha profiles in different layers of intracortical measurements (Bollimunta et al., 2008, 2011). If different alpha generators were indeed negatively coupled, the up-modulation in one (i.e., by NIBS) could lead to a down-modulation of the other. The single trial, cross-validated CSP filtering procedure extracts components and maximizes a stimulation-induced change in alpha power, facilitating the revelation of a spatially distinct alpha component that shows a decrease in power. Crucially this decrease captured in different components may be caused by different underlying mechanisms: it may capture an antagonistic decrease during in-phase tACS up-modulation, a decrease due to a potentially disturbing effect of anti-phase tACS or a homeostatic rebound after an up-regulation during tACS as a (meta)-plastic effect (Abraham, 2008; Gundlach et al., 2017). Our optimization algorithm (CSP) indicated consistently a decrease in the amplitude of alpha oscillations, regardless of the relationship between the phase of tACS and the phase of the ongoing alpha oscillations. This finding may thus suggest that the effect of any briefly applied tACS for occipital alpha generators, be it in- or anti-phase may be rather disruptive in its nature.

To our knowledge, we are the first to utilize CSP filtering for the analysis of tACS effects and thus to obtain neural activity selectively tuned to show a decrease or an increase of alpha oscillations following tACS. Although CSP is mainly applied in BCI paradigms to differentiate particular activation patterns, which usually correspond to different anatomical regions (e.g., left or right motor imaginary corresponding to the activation of the right or left motor cortex), this method also can be useful for discriminating between activity in the same spatial region, which was previously shown for alpha oscillations with standard and deviant visual stimuli (Tugin et al., 2016). In our study, CSP was used to provide spatial filters maximally discriminating activity between the periods with and without stimulation. This in turn allowed the contribution of stimulation-related neural changes to be maximized while attenuating irrelevant neural activity typically masking effects of stimulation in the sensor space. As previously mentioned, tACS always affects a broad region of neural populations, and thus studying its influence based only on data from a single or few channels is only an approximate simplification. Such approaches lead to a significant reduction of the observation space, the omission of region-specific dynamics, and raises the impact of noise and volume conduction in the data. Therefore, to perform a deeper, more comprehensive, and more extensive investigation of the respective research question, CSP and other spatial filtering methods should be considered for the analysis of tACS effects and brain stimulation effects in general. Importantly, the modulatory effects of tACS were primarily limited to the occipito-parietal regions—areas targeted with tACS in our study. Such spatial distribution of the observed effects challenges the possibility that the effects of tACS might have been due to the stimulation of the scalp (Vöröslakos et al., 2018; Asamoah et al., 2019). In this case, we would expect attenuation of alpha/mu oscillations over the sensorimotor areas, which was not the case here. Even though recent work strongly favors the direct modulation of neural activity by tACS (Kasten et al., 2019; Krause et al., 2019; Negahbani et al., 2019; Vieira et al., 2019) the contribution of various sources such as peripheral or retinal stimulation is currently controversially discussed. Given the here found phase-independent stimulation effects, we cannot rule out a potential contribution of general stimulation effects. Future work will have to disentangle the contribution of different mechanisms to overall effects.

One limitation of our study is that our design may be suboptimal in promoting plastic changes in neural activity developing during the time of the stimulation. Using a purely event-related design, the stimulation phase varied randomly between in-phase and anti-phase with ongoing alpha oscillations. If tACS were able to lead to online entrainment of ongoing neural oscillations as a potential prerequisite for offline effects, one could hypothesize that this effect would be more pronounced when the same phase-orientation (“in” or “anti”) were utilized over the whole duration of the stimulation block or experiment (Deans et al., 2007; Fröhlich and McCormick, 2010; Ozen et al., 2010; Helfrich et al., 2014). Varying the phase relationship across trials might have interfered with an effect that relies on accumulating over time and may have masked the potential effects of tACS.

Another limitation concerns the sampling of the phase space phase to evaluate a potential relationship between mu-alpha-band activity and applied tACS. While we varied signals to examine the two most-extreme relationships (in phases vs. in opposite phase) and focused our phase extraction-, stimulation- and analysis-regimes to capture and modulate parieto-occipital alpha-generators, this may not capture the richness of the underlying dynamics that may stem from an interplay of thalamic and multiple cortical alpha generators (Bollimunta et al., 2008; Meij et al., 2016). Given the cost of extensively sampling the whole parameter space, promising new approaches like Bayesian sampling (Lorenz et al., 2019), may help to draw a more complete picture of underlying dynamics. These approaches may also help to elucidate the impact of different stimulation parameters like the amplitude of the applied tACS current which varies between studies (for an overview see Strüber et al., 2015; Veniero et al., 2015) and may likely affect stimulation outcomes. While we used a stimulation intensity that is comparable to previous studies (Helfrich et al., 2014; Strüber et al., 2015; Gundlach et al., 2016, 2017), we cannot rule out that phase-dependent stimulation effects may potentially be measurable for higher stimulation intensities.

Closed-loop tACS and, in general, adaptive NIBS in comparison to conventional stimulation protocols have several advantages, which could be potentially beneficial for the whole brain stimulation research field and transform it into a more reliable and clinically applicable approach. As mentioned by Zrenner et al. (2016), these advantages include: personalized neuromodulation to decrease inter-individual variability of effects, analysis of network reorganization dynamics, such as during stroke, for instance, to aid rehabilitation and target as well as specifically modify potentially different plasticity patterns. One of the main challenges impeding the development of closed-loop tACS, however, is the fact that the analysis of online effects during stimulation is compromised by massive stimulation-induced artifacts. In principle, the signal to be analyzed and modulated (e.g., a signature of alpha oscillations measured in the EEG) is overwritten by the tACS signal, which is several magnitudes larger but covers the same spatial and temporal space. Thus, substantial efforts in recent studies have been directed to the development of artifact elimination methods using different techniques and experimental protocols (Witkowski et al., 2016; Noury and Siegel, 2017; Kasten et al., 2018; Kohli and Casson, 2019). However, intermittent stimulation allows one to follow another approach while still based on adaptive principles. By studying the immediate after-effects in intervals between periods of stimulation without artifacts, such studies may contribute towards exploring related online effects and enhance the understanding of tACS effects and mechanisms in general. One prominent recent study with phase-locked closed-loop stimulation was presented by Mansouri et al. (2018). By using intermittent stimulation with very short (5 ms) square-wave pulses and an artifact removal procedure using a spline interpolation (Waddell et al., 2009), they were able to extract the artifact-free EEG signal and thus control for the actual phase locking of delivered stimulation and ongoing oscillation in alpha and theta bands. Such approaches may help to elucidate the role of adaptive NIBS on brain activity and ultimately the role of brain activity on cognition, perception, and behavior in general.

**Figure 7:**
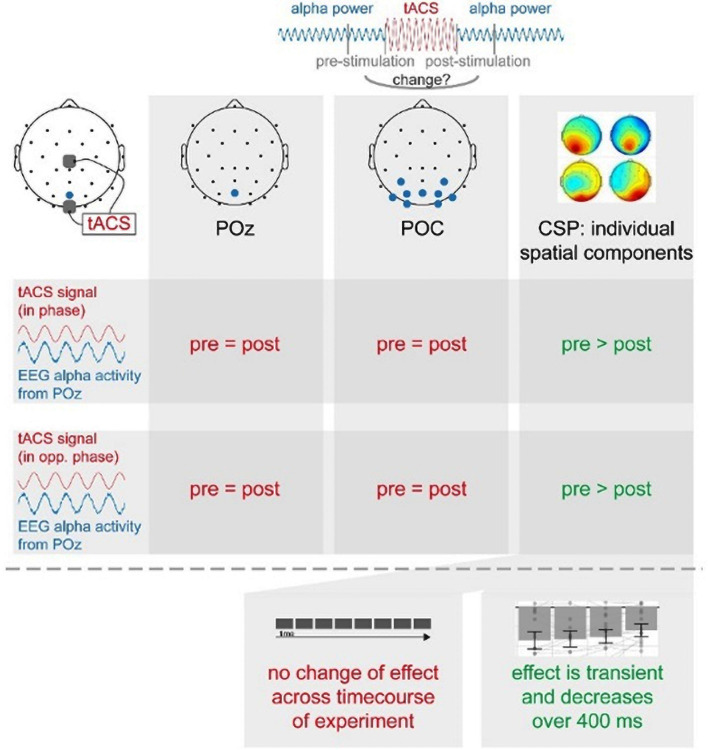
Summary of main experimental findings.

In summary (see Figure 7), we found that short-time intermittent tACS applied over occipital regions (Cz and Oz), as used in previous studies, induces a transient suppression of occipital alpha generators, leading to a decrease in alpha power in spatially specific components centered over a parieto-occipital region. This effect was independent of the phase relationship between the tACS signal and alpha oscillations. This suggests to us that these offline effects of short-timed intermittent tACS are not explainable by entrainment alone but rather require neuroplastic changes or a transient disruption of neural oscillations. These effects were only visible in individual spatial alpha components, but not in a broad occipital cluster or pre-selected electrode. Our study thus supports the notion that the response to tACS differs inter-individually and that even intra-individual effects are shaped by the interplay between different alpha generators. This favors stimulation protocols as well as analysis regimes exploiting inter-individual differences to more efficiently induce as well as more reliably reveal otherwise hidden stimulation effects and thereby comprehensively study the effects and the underlying mechanisms of tACS.

## Data Availability Statement

The datasets generated for this study are available on request to the corresponding author.

## Ethics Statement

The studies involving human participants were reviewed and approved by the Ethical Committee at the Medical Faculty, Leipzig University, under the protocol “Modulation neuronaler Oszillationen mittels transkranieller Wechselstromstimulation und ihr Effekt auf die somatosensorische Wahrnehmung,” 12.08.2014, Reference number: 218-14-14072014. The patients/participants provided their written informed consent to participate in this study.

## Author Contributions

GZ, CG, AV, and MB: conceptualization. GZ and CG: data curation. GZ, CG, and VN: formal analysis. AV and MB: funding acquisition. GZ, CG, and VN: investigation. GZ, CG, VN, and MB: methodology. GZ and CG: project administration, visualization, and writing—original draft. MB and AV: resources. GZ, CG, VN, and MB: software. VN, AV, MB, and CG: supervision. GZ, CG, and VN: validation. GZ, CG, VN, AV, and MB: writing—review and editing.

## Conflict of Interest

The authors declare that the research was conducted in the absence of any commercial or financial relationships that could be construed as a potential conflict of interest.
