# Supplementary material for: Transient Amplitude Modulation of Alpha-Band Oscillations by Short-Time Intermittent Closed-Loop tACS
Source: Front Hum Neurosci. 2020 Sep 4;14:366. doi: 10.3389/fnhum.2020.00366 (PMC7500443; doi:10.3389/fnhum.2020.00366)

Supplementary Material

SUPPLEMENTARY METHODS

Closed-loop model

The general model scheme is represented in Figure 1A. The model is implemented with 3 modes of functioning: “Online”, “Offline”, “Record-only” / “Stimulation-only”. In “Online” mode (Figure 1C) the system functions in a state required for adaptive stimulation with short interval cycles (e.g., 1 s) and incorporates: import of EEG signals (pre-stimulation interval), estimation of optimal parameters (e.g., phase shift) and calculation of stimulation signal, computation of required optimization time, compensation of optimization and transduction delays, and transmission of stimulation signal (stimulation interval) through a NI DAQ card to the stimulator device input (BNC port). A typical cycle also contains a post-stimulation interval for analysis of effects and inter-trial interval. The “Offline” mode was used for the testing of phase prediction methods, while the “Record-only” and “Stimulation-only” modes can be used for continual recording of data and for continual stimulation with fixed parameters. All computations were performed on Lenovo P70 (Intel i7 OctaCore 2.6GHz, 16Gb RAM, Lenovo Group Limited, Beijing, China).

Phase prediction, delay estimation and compensation

For phase prediction we utilized the same Hilbert-based approach as tested and validated in our previous study (Zarubin et al., 2018). This method is based on the assumption that with short time intervals we can consider phase dynamics to be quasi stationary, which allows us to perform phase prediction by the extraction phase information from a current (pre-stimulation) interval in order to forecast phase dependent stimulation for the following interval. Importantly, we only used a predefined part of the pre-stimulation interval for phase extraction (extraction interval), which is controlled by a parameter and was settled to the last quarter (250 ms) of the whole interval (Figure 1D). For the extraction interval, a phase lag between estimated phase value and the alpha phase on this interval should be minimized to achieve the best prediction. For this optimization we used Hilbert transformation (after FIR filtering, 100 ms length) to obtain instantaneous phase values and iterative search across sine waves with different phases. Euclidean distance (L2 norm) for vectors of instantaneous phase between extraction interval and various generated sine waves was the criterion for minimization -in case of “in-phase” relation, and maximization in case of “anti-phase” relation. Transduction and optimization delays were estimated and compensated with the same procedure as validated in (Zarubin et al., 2018). For the chosen phase prediction method (Hilbert-based), the average value of optimization delay across all subjects was 15±2.4 ms and average transduction delay was 72 ms.

Calculation of Common Spatial Patterns

The motivation for the application of spatial filters to investigate effects of stimulation is driven by the fact that while effects of tACS have a relatively broad distribution, they still influence a rather well-defined neuronal population. The extraction of neural activity from such a population, however, is hampered by volume conduction, which leads to the EEG recording reflecting many neuronal populations with overlapping fields. Thus, in order to extract the EEG activity most susceptible to

stimulation, we use spatial filtering that maximizes/minimizes the difference in power between the reference and post-stimulus intervals. We can accomplish this using spatial filters that maximally discriminate between on and off stimulation intervals. One of the established and efficient methods for extraction of modulated brain activity is the Common Spatial Patterns (CSP) approach, which was introduced in (Blankertz et al., 2008) for single-trial analysis and is commonly used in brain-computer interface (BCI) applications to differentiate between classes of brain activation. Moreover, CSP has been successfully applied previously for the extraction of alpha band activity sensitive to the processing and discrimination of standard and deviant visual stimuli (Tugin et al., 2016).

We utilized CSP to obtain data from parietal-occipital regions to investigate possible differences between pre- and post-stimulation activity for alpha band oscillations in visuo-occipital components. Firstly, channels affected by sustained electrical current from neighboring tACS electrodes were rejected via EEGLAB functions and visual inspection. Then we extracted and merged all remaining data from pre- and post-stimulation intervals in two matrices (S_{pre} and S_{post}). The post-stimulation data collection began 80 ms after stimulation offset, the arbitrary 80 ms shift was applied to avoid possible stimulation-related artifacts. Further, the data intervals were band-pass filtered with a frequency range of 7 to 14 Hz with 4th order Butterworth zero-phase filter to prevent edge effects on borders caused by baseline shifts during experiment. Next, we constructed two new matrices (S'_{pre} and S'_{post}) based on S_{pre} and S_{post} by cutting respectively the last and first 100 ms from each interval, of the original matrices, to avoid the influence of filtering edge effects would have on further analysis. Then, following the scheme described earlier (Time intervals, Figure 1E), 500 ms of data preceding the stimulation was used as the pre-stimulation interval and 500 ms following the stimulation as the post-stimulation data. Further, covariance matrixes (C_{pre},C_{post}) for pre- and post-stimulation states were determined (1):

$$C_{st} = \frac{1}{n-1} S'_{st} {S'}^{T}_{st}, (1)$$

$$st: [pre, post],\ n - \text{ number of trials},\ C_{st} \in R^{m \times m}, m - \text{ number of channels}$$

Afterwards generalized eigenvalue decomposition of (C_{pre},$C_{pre} + C_{post}$) was computed (2):

$$C_{pre}\mathrm{W} = \Lambda\, C_{post} W, (2)$$

$$W \in R^{m \times m} - \text{ matrix of eigenvectors},\ \Lambda - \text{ matrix of eigenvalues}$$

Eigenvectors w_j ($j = 1{:}\,m$) of the decomposition are CSP filters, where eigenvectors corresponding to higher eigenvalues maximize pre-stimulation activity (i.e. a decrease in alpha power from pre- to post-stimulation period). Eigenvectors with the lower eigenvalues maximize post-stimulation activity (i.e. an increase in alpha power from pre- to post-stimulation period). Vectors of the inverse matrix W^{-1} represent spatial patterns. When the CSP filters were computed, for every subject from the same order of filters we selected one filter maximizing pre-stimulation alpha power over post-stimulation power (CSP(pre): w_j) and one maximizing post- over pre-stimulation alpha power (CSP(post): w_{m-j+1}). Selection of order j was performed by analyzing the topographies of the CSP filters (with the inverse matrix W^{-1} and the topoplot() function in EEGLAB). Here the first filter was chosen for which the spatial pattern showed a parietal-occipital topography. This allowed us to consider specifically alpha oscillations from regions targeted with tACS. Importantly, to avoid overfitting, we implemented a cross validation procedure for every trial. For each single trial, spatial filters were first constructed based on data, not from that trial, but from all other trials (i.e., a leave-one-out procedure). Then, the selected CSP filter was normalized by its standard deviation (3) and applied to the trial to obtain the projected component $P_{pre/post,i}$ of a current trial X_i (4):

$$w_j' = w_j/std(w_j), (3)$$
$$P_{pre,i} = w_j' X_i^T,\ P_{post,i} = w_{m-j+1}' X_i^T, (4)$$
$$X_i^T \in R^{m \times k}, i \text{ – number of a current trial}, k \text{ – length of trial}, \mathrm{k} = 1000$$

Crucially, the CSP estimation and selection was thus independent of the data for which these filters were then adopted. Finally, the obtained component $P_{pre,i}$ of all trials were merged and used in the further analysis as CSP(pre) data, whereas the components $P_{post,i}$ were merged and used as CSP(post) data.

Spatially-specific and individualized analysis on the basis of CSP components

Spatial topographies for exemplary subjects and average spatial topographies for CSP(pre) and CSP(post) are shown in Figure 3. Patterns show weights of the inverse matrix of CSP filters. Because the polarity of the distribution is arbitrary, the topographies were normalized by changing the sign to positive in case of negative weights for the patterns at the occipital-parietal area. For the estimation of the average topographies, the patterns of each subject were first normalized by the standard deviation of all channel weights. These normalized patterns could then be averaged. While individual topographies showed some variations in the precise distribution, the average topographies clearly represented the occipito-parietal area, which was our targeted region. In addition, components were constructed with narrow band-pass filtering (7–14 Hz), thus, the data obtained with CSP components mainly extracts alpha oscillations.

Supplemental Figure 1. Single subject and average CSP topographies. For each subject as well as the average of all subject topographical distributions of the inverse matrix of the CSP filters are shown for CSP(pre) on the left and CSP(post) on the right.

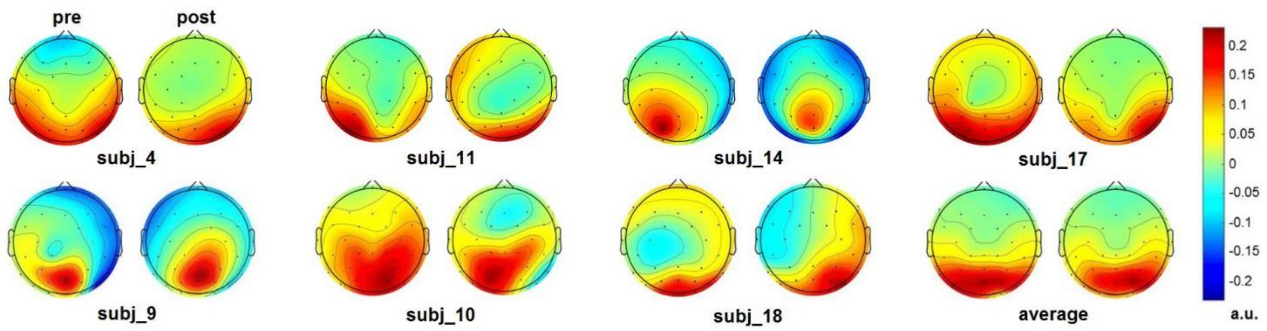

Supplement: Supplementary file 1 [file Data_Sheet_1.PDF]
